# Evaluating Flight Crew Performance by a Bayesian Network Model

**DOI:** 10.3390/e20030178

**Published:** 2018-03-08

**Authors:** Wei Chen, Shuping Huang

**Affiliations:** 1 School of Naval Architecture, Ocean and Civil Engineering, Shanghai Jiao Tong University, Shanghai 200240, China; 2 State Key Laboratory of Ocean Engineering, School of Naval Architecture, Ocean and Civil Engineering, Shanghai Jiao Tong University, Shanghai 200240, China

**Keywords:** flight crew, Bayesian Network, Delphi technique, leaky noisy MAX model

## Abstract

Flight crew performance is of great significance in keeping flights safe and sound. When evaluating the crew performance, quantitative detailed behavior information may not be available. The present paper introduces the Bayesian Network to perform flight crew performance evaluation, which permits the utilization of multidisciplinary sources of objective and subjective information, despite sparse behavioral data. In this paper, the causal factors are selected based on the analysis of 484 aviation accidents caused by human factors. Then, a network termed Flight Crew Performance Model is constructed. The Delphi technique helps to gather subjective data as a supplement to objective data from accident reports. The conditional probabilities are elicited by the leaky noisy MAX model. Two ways of inference for the BN—probability prediction and probabilistic diagnosis are used and some interesting conclusions are drawn, which could provide data support to make interventions for human error management in aviation safety.

## 1. Introduction

By very nature, human beings make mistakes [1]. Hence, it comes as no surprise that 70% to 80% of aviation accidents involve human errors [2]. Of these human-error-related accidents, approximately 60% involved flight crew errors [3,4]. Crews conduct the flight mission directly and should correspond to various external threats properly and in time [5]. Therefore, excellent flight crew performance is regarded as the key element to ensure continuing safe and reliable air transportation services for the public.

In order to improve flight crew performance, quite a lot of research has been done by governmental and non-governmental organizations, of which a key component is Crew Resource Management (CRM) training [6,7]. As the sixth generation of CRM training developed, Threat and Error Management (TEM) emerged as well. On the basis of this model, Line Operations Safety Audits (LOSA) collects safety data in normal flight operations and provides a quantitative view of external threat and flight crew error [8,9]. LOSA collects data just during the normal operation of each flight, but detailed behavior information that is needed for flight crew performance evaluating may be available in accident reports. Furthermore, quantitative causal relations are usually limited. The relationships between causes or influence factors appear to be complicated with great uncertainty.

Faced with the problem of data scarcity, the Bayesian Network (BN) model is introduced in this paper, which could make inference from incomplete, imprecise and uncertain knowledge [10,11]. In the BN model, all related factors could be presented with the dependence relations, which reflects the hierarchical nature of influence domains [12]. Moreover, multidisciplinary sources of objective and subjective information could be integrated. As a methodology that has been used in analysis of human failures by many researchers, the BN model provides an important supplement in describing flight crew performance both qualitatively and quantitatively.

The BN modeling process and application in the field of aviation safety (especially about inspection and aviation maintenance) have been discussed in the authors’ previous studies [13,14]. Nevertheless, only two states are set for most nodes in the network due to the limitations of the noisy OR gates and the recursive noisy OR rule, which are used to generate CPTs (Conditional Probability Table). In this paper, the noisy leaky MAX model that could handle multiple states of nodes is used instead.

The rest of this paper is organized as follows. Section 2 gives a brief introduction of methodologies that are applied in this research, including the BN model, the noisy leaky MAX model, and the Delphi Technique. In Section 3, a BN model for flight crew performance is constructed based on the analysis of 484 aviation accidents and CPTs are elicited. Section 4 presents sensitivity analysis of the influence factors. Conclusions and further research work are summarized in Section 5.

## 2. Materials and Methods

### 2.1. Bayesian Network

**Figure 1:**
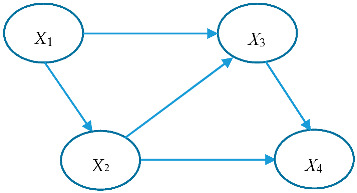
A Bayesian Network (BN) sample.

The BN model, proposed by Pearl in 1988, is a probabilistic graphical model that consists of a set of nodes, representing random variables with finite states, and edges, representing their conditional independencies in a Directed Acylic Graph (DAG) [15]. The nodes with edges directed into them are child nodes (e.g., *X*_2_ in Figure 1) and the nodes from which the edges depart are parent nodes (e.g., *X*_1_ in Figure 1) and nodes without arches directed into them are root nodes (*X*_1_). The causal dependence between variables is expressed by the structure of nodes, which gives the qualitative part of causal reasoning in a BN. The relationship between variables and the corresponding states are presented in the form of CPT attached to each node, which constructs the quantitative part. Bayesian inference, D-separation, and chain rule are key concepts in BN modeling [14].

#### 2.1.1. Conditional Probabilities and Bayes Theorems

The fundamental formulae of Bayesian inference is shown as below:

(1) $$P(B|A)=\frac{P(A|B)P(B)}{P(A)}$$

- *B* refers to a specific hypothesis or a set of hypothesizes;
- *A* refers to an observed evidence *E*;
- P(B) refers to prior probability before getting the evidence *E*;
- P(A|B) refers to the probability that *E* presents in every situation of *B*;
- P(B|A) refers to the posterior probability after getting the evidence *E*; and,
- P(A) is the marginal probability of *A*.

For each node *A* in BN, apart from marginal nodes, there is a CPT. $P(A|B_{1},\ldots ,B_{n})$ is decided by one’s parent nodes $B_{1}$, …, $B_{n}$.

#### 2.1.2. D-Separation and Conditional Independence

**Figure 2:**
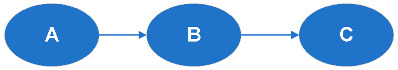
Serial Connection.

**Figure 3:**
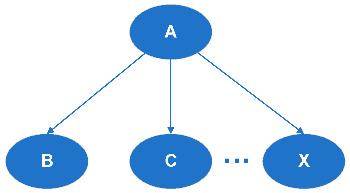
Diverging Connection.

**Figure 4:**
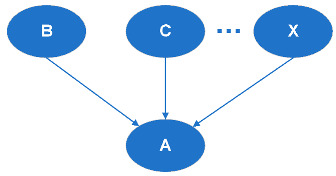
Converging Connection.

There is another fundamental concept in BN called D-separation. D-separation means the blocking of the transmission through a casual network. Its mathematical definition is as follows: “Two variables A and B in a causal network are D-separated if for all paths between A and B there is an intermediate variable V such that either the connection is serial or diverging and the state of V is known or the connection is converging and neither V nor any of V’s descendants have received evidence.” 3 types of connections in BN applying D-separation are shown as below:

(a) Serial Connection (see Figure 2)
  When there is evidence given about B, the communication between A and C is blocked.
(b) Diverging Connection (see Figure 3)
  When there is evidence given about A, the communication between B and C is blocked.
(c) Converging Connection (see Figure 4)
  When there is evidence given about A, communication is blocked between its parent nodes.

In use of D-separation, child nodes only relate to their parent nodes. In the Bayesian calculus, D-separation is reflected in the concept of conditional independence. It can be formulated as:

*P*(*A*|*B*) = *P*(*A*|*B*,*C*) (2)

It means whether there is evidence about event B or not, no knowledge regarding event A will change the probability of event C.

Using Chain Rule and D-separation, the following is valid.

*P*(*A*_1_,*A*_2_,…,*A*_n_) = *P*(*A*_1_)*P*(*A*_2_|*A*_1_)…*P*(*A*_1_|*A*_2_,…,*A*_n−1_) (3)

A Bayesian Network is a representation of the joint probability distribution over all of the variables represented in the DAG [16]. The marginal and the conditional probabilities for each node of the network are computed by the chain rule.

Then, according to the Bayes rule, probabilistic inference is processed, which lies in two-way reasoning: diagnostic (bottom-up) and predictive (top-down). Given an evidence about the possible states of a subset of the variables of the network, the probability of occurrence of some events can be calculated.

Therefore, by probabilistic inference in Bayesian Networks it is possible to make inference of unobserved nodes. The posterior probability obtained from inference in BN (diagnosis or prediction) is of great importance for updating the network and decision making.

#### 2.1.3. BN Modeling

**Figure 5:**
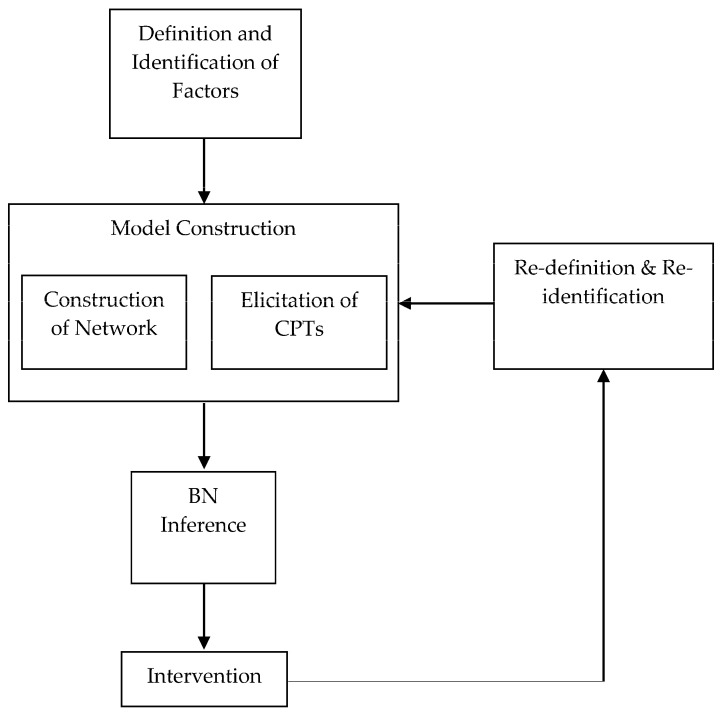
BN Modeling Process.

BN modeling steps are summarized as follows (see Figure 5) [13,14]:

- Step 1: Definition and identification of influence factors.
- Step 2: Construction of the BN model, which includes development of the network and elicitation of the CPTs. The dependence relations of the influence factors are modeled in a BN diagram and quantitative dependency is specified by eliciting the CPTs.
- Step 3: BN inference and finding key factors.
- Step 4: Intervention. Corresponding to the key factors, some measures should be taken to improve the safety condition.
- Step 5: Re-definition and re-identification. The process should be constant and the model should be improved continuously.

### 2.2. Noisy MAX and Leaky Noisy MAX

Generally, CPTs can be elicited from databases or human experts’ judgments [17]. However, it is challenging and doubtful work to obtain conditional probabilities in a large-scaled network directly due to the exponential growth of the number of parameters [18,19]. Some Canonical interactions, such as the noisy OR gates, are developed to solve this problem, which take advantage of independence of causal interactions and offer a logarithmic reduction of the number of parameters required to specify a CPT.

Generally, for complete conditional distribution of *n* binary predecessors, 2*^n^* parameters are required. But, when using the noisy OR gates, the number of parameters required changes to only *n*, which is the number of predecessor nodes. Therefore, the complexity of knowledge acquisition is reduced [20]. But in order to apply the OR model in practice, the network builders should verify that the noisy OR gates cannot be used if the variables involved in the network are not Boolean [21].

To solve this problem, Henrion first proposed the generalization of the OR model to multi-valued variables [22] and Díez formalized Henrion’s model, coined the term “MAX gate” [18,21,23].

#### 2.2.1. Noisy MAX Model

In this paper, random variables (nodes) are represented by upper-case letters (e.g., *Y*) and their values indexed by lower-case letters (e.g., *y*_1_). In the noisy MAX model, child node *Y* takes on *i* possible values denoted by *Z_i_* (*Y* and the *Z_i_* s share the same domain), and $Pa(Y)=\left\{X_{1},\ldots ,X_{n}\right\}$ usually stand for the causes of *Y*. $c_{z_{i}}^{x_{i}}$ means the probability that parent node *X_i_*, when taking the value *x_i_*, results in $Y=z_{i}$. The parameters for a link *X_i_*→*Y* are:

(4) $$c_{z_{i}}^{x_{i}}=P(z_{i}|x_{i})$$

or, equivalently,

(5) $$c_{y}^{x_{i}}=P(Z_{i}=y|x_{i})$$

In the noisy MAX model, P(Y≤y|X) for all of the values *y* and all of the configurations *X* should be computed at first in order to obtain the CPT:

(6) $$\begin{array}{l} P(Y\leq y|X)=\sum_{z|f_{MAX}(z)\leq y}\prod_{i}c_{z_{i}}^{x_{i}}\\ =\sum_{z_{1}\leq y}\cdot \cdot \cdot \sum_{z_{n}\leq y}\prod_{i}c_{z_{i}}^{x_{i}}=\prod_{i}\left(\sum_{z_{i}\leq y}c_{z_{i}}^{x_{i}}\right) \end{array}$$

where

(7) $$f_{MAX}(z)=\max(z_{1},\ldots ,z_{n})$$

Then, the CPT can be obtained as follow:

(8) $$P(y|X)=\{\begin{array}{cc} P(Y\leq y|X)-P(Y\leq y-1|X) & y\neq y_{min}\\ P(Y\leq y|X) & y=y_{min} \end{array}$$

#### 2.2.2. Leaky Noisy MAX Model

Like any other knowledge representation model, the BN model is never complete as it could not model every possible cause of an effect [24]. To allow for this in the noisy MAX model, an additional variable *Z_L_*, called the leaky variable, can be introduced to represent set of causes that are not modeled explicitly. The MAX model which takes leaky probabilities into account is termed as the leaky noisy MAX model. In the model, $dom(Z_{L})=dom(Y)$, which implies that *i* possible leaky parameters $c_{y}^{L}$ are needed to compute the conditional probabilities:

(9) $$c_{y}^{L}=P(Z_{L}=y)$$

Similarly to the case of noisy MAX model, P(Y≤y|X) should be computed according to the following formula before CPT elicitation:

(10) $$\begin{array}{l} P(Y\leq y|X)=\sum_{z}\prod_{i|X_{i}\in X}P(z_{i}|x_{i})\sum_{z_{L}|f_{MAX}(z,z_{L})\leq y}P(z_{L})\\ =(\sum_{z_{L}\leq y}c_{z_{L}}^{L})\cdot \prod_{i}\left(\sum_{z_{i}\leq y}c_{z_{i}}^{x_{i}}\right) \end{array}$$

Define an accumulative vectorial parameter,

(11) $$C_{y}^{L}=\sum_{z_{L}\leq y}c_{z_{L}}^{L}$$

then Equation (10) becomes

(12) $$P(Y\leq y|X)=C_{y}^{L}\cdot \prod_{i}C_{y}^{x_{i}}$$

In the end, each value of CPT can be obtained as Equation (8).

### 2.3. The Delphi Technique

When hard data is unavailable or too costly to obtain, the Delphi technique is a useful tool to “obtain the most reliable consensus of opinion of a group of experts” [25].

When conducting a Delphi survey, a panel of experienced experts who are very familiar with the specific subject in the area are selected. Questionnaires were transmitted and filled in by each expert strictly individually. After compiling all of the data gathered from the experts, feedbacks of responses are exchanged with the panelists in order to achieve a consensus. In the technique, information can be exchanged via e-mail, mail, FAX, which may avoid counterproductive discussions and digressions in face-to-face group discussions.

When evaluating the flight crew performance, not all detailed behavior information needed can be available. Opinions from experts on this domain plays an important part of the data sources, other methods such as the Classical Method, are applicable to deal with experts opinions [26,27,28].

## 3. The Flight Crew Performance Model

### 3.1. Network Construction

**Figure 6:**
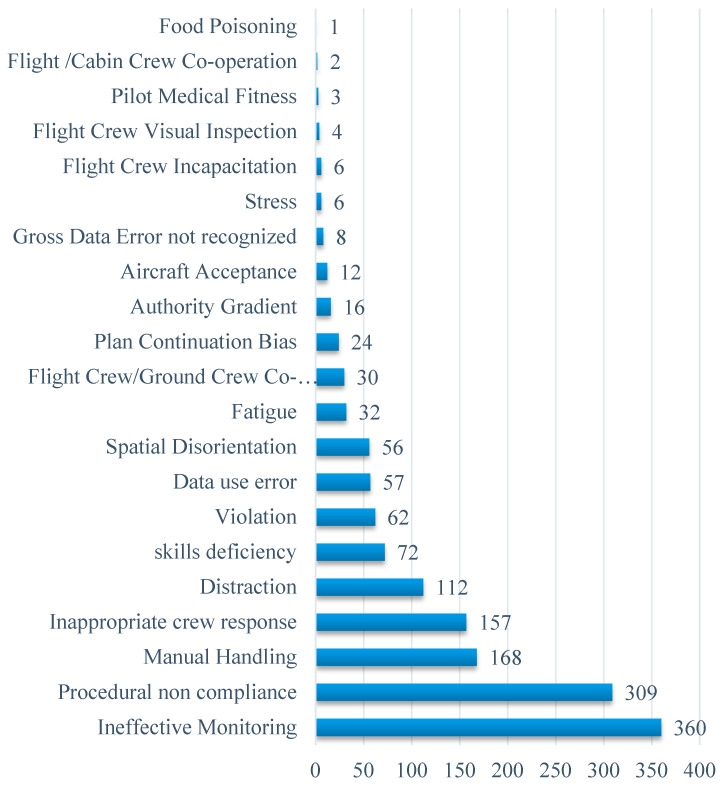
Causes Related to Flight Crew Involved in 484 Aviation Accidents.

In this study, detailed information of 484 aviation accidents related to human factors occurring from 1999 to 2012 are gathered from the website www.skybrary.aero and the causes of each accident are analyzed according to the final report. The frequencies of various causes involved in all of these accidents are counted (see Figure 6), and the top 5 causes are ineffective monitoring, procedural non compliance, manual handling, inappropriate crew response, and distraction.

**Figure 7:**
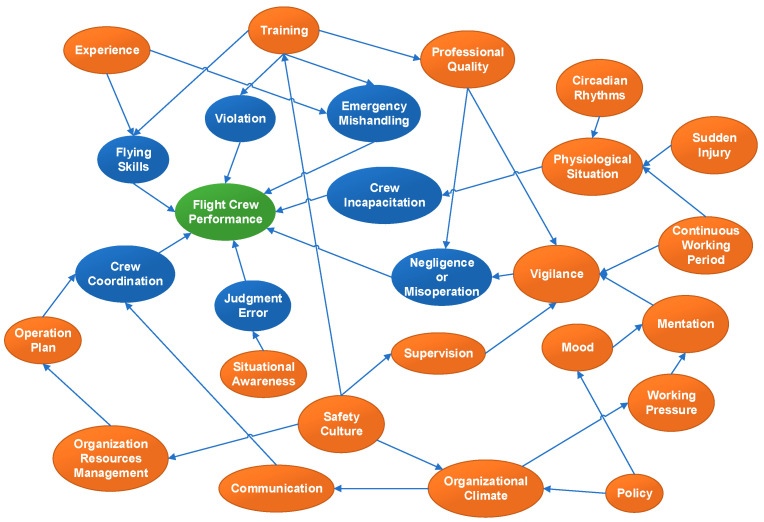
Flight Crew Performance Model.

Based on the analysis of aviation accidents, the most influential factors of flight crew performance are selected. Then, the relations between factors are defined by experts in the field of aviation safety and shown graphically in a network (see Figure 7), which is termed as the “Flight Crew Performance Model”.

### 3.2. Data Collection

Data collection has always posed a very serious problem when it comes to human factors. As accident reports cannot record concrete information about every node in the model, it is quite difficult to elicit CPT just based on reports. In this paper, two methods are applied to collect data in different probability scenarios.

#### 3.2.1. Marginal Probability Scenario

Data fusion from related literatures and reports are used in marginal probability scenarios. Take the node Experience for example to illustrate this process. According to the qualification requirements of Federal Aviation Administration (FAA), 1500 h of pilot flight time are needed to hold an Airline Transport Pilot License (ATPL) [29]. In this paper, three states are set for the node Experience: poor, normal, and rich, which correspond to “pilot flight time < 1500 h”, “1500 h < pilot flight time < 5000 h” and “pilot flight time > 5000 h”. According to 434 related records available for 484 aviation accidents, the marginal probabilities of this node are calculated and shown in Table 1.

#### 3.2.2. Conditional Probability Scenario

In conditional probability scenarios, survey is conducted to obtain the original parameters, which are used to generate conditional probabilities by noisy Leaky MAX model. The survey is carried out through the process known as the Delphi Technique. As introduced above, this technique is a methodical interactive procedure that completely relies on the knowledge of a panel of experts whose duty is to predict an outcome which is normally achieved through the goal of consensus building without bringing the experts face to face.

There are two types of questions that are posed for each node in the survey, corresponding to the modeled causes and unmodeled causes. Take the node Organizational Climate in Figure 7 as example, the question amounted to:

- What is the probability that “Safety Culture = good” results in “Organizational Climate = good” in the absence of the cause “Policy = good”?
- What is the leaky probability of the unmodeled causes result in “Organizational Climate = good” in the absence of the causes “Safety Culture = good” and “Policy = good”?

The original values were gathered and processed according to the Absolute Probability Judgment (APJ) rule. Then, the CPTs are computed based on these values according to the Leaky Noisy MAX Model stated above.

## 4. Discussions

In this research, the conditional probabilities are calculated and inference is conducted with the use of software GeNie. After BN modeling, sensitivity analysis of probabilities can be made by giving different subsets of evidences. Also, two-way reasoning: bottom-up diagnostic and top-down predictive could be performed on the basis of BN probabilistic inference.

### 4.1. Key Factors

By setting states of each influence factor, the posteriors of the target node Flight Crew Performance are compared in Table 2 and the “Increased Percent” measures the influences of different influence factors on crew performance.

As shown in Table 2, the top 5 key influence factors are flying skills, vigilance, emergency mishandling, safety culture and crew coordination. One interesting point is that safety culture, which is an invisible element, ranks fourth among all of the nodes. Safety culture is defined as the product of individual and group values, attitudes, perceptions, competencies, and patterns of behavior about organization’s health and safety management [30]. Safety culture penetrates into all aspects of the organization and has great impact on everybody including the flight crews.

### 4.2. Bottom-up Diagnostic

**Figure 8:**
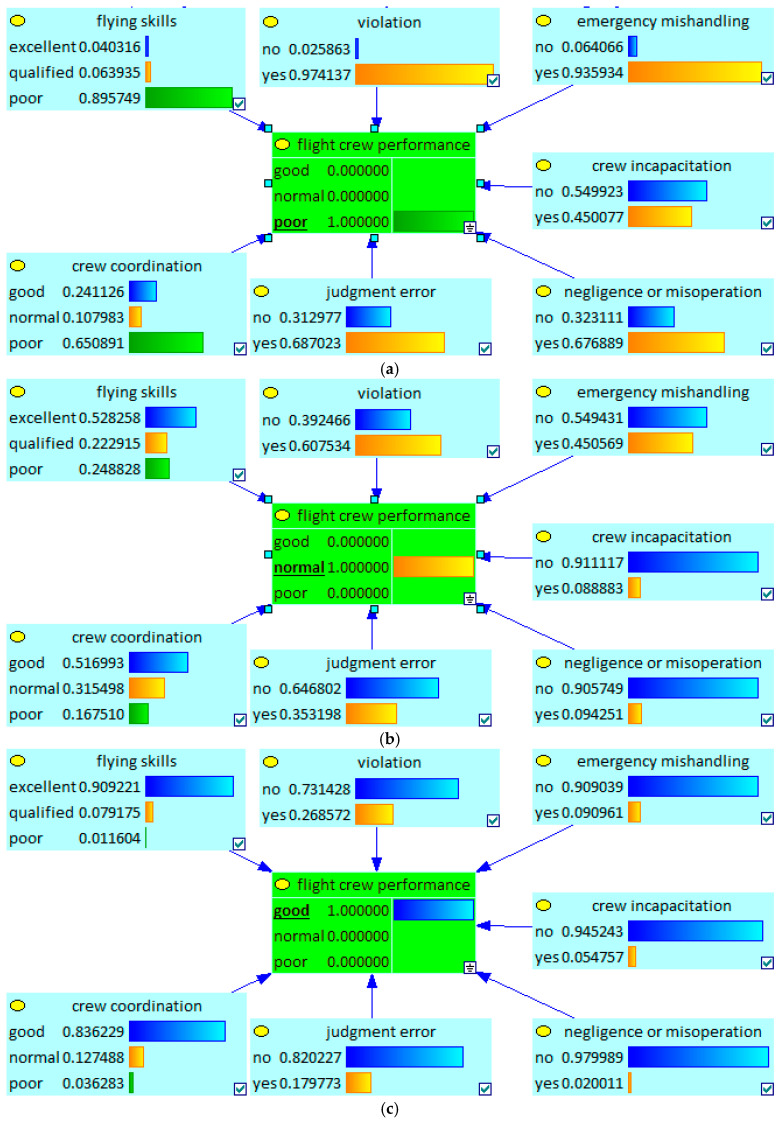
Bottom-up Diagnostic example for the Target Node. (**a**) The computation results about parent nodes when the node Flight Crew Performance is set as “poor”; (**b**) the computation results about parent nodes when the node Flight Crew Performance is set as “normal”; (**c**) the computation results about parent nodes when the node Flight Crew Performance is set as “good”.

In Section 4.1, the process of finding key factors is a kind of top-down predictive reasoning. If evidence about the target node or the child nodes are given, diagnosis can be performed by computing the probabilities of their parent nodes. Take the node Flight Crew Performance as an example, three states “good”, “normal”, and “poor” are set, respectively, and the computation results about parent nodes are shown in Figure 8. When Flight Crew Performance turns out poor, the output of node Violation and node Emergency Mishandling are “yes = 0.974137” and “yes = 0.935934”, respectively, which is significantly higher than other parent nodes. It could be inferred that the most possible causes are violation and emergency mishandling when the flight crews do a bad job. But when the state of Flight Crew Performance is set as “good”, the nodes Negligence or Misoperation and Crew Incapacitation become more attractive, which may indicate that these two factors play more important roles under this situation.

### 4.3. Sensitivity Analysis

Sensitivity analysis is a general technique for studying the effects of parameter inaccuracies on the output of a mathematical model. In Section 4.1, sensitivity analysis is applied to compare the importance of different factors. The mathematical function and analysis based on it will be shown in this part.

When carrying out sensitivity analysis, the sensitivity function can be used to express the sensitive change in posterior probability of the target query [31]. If *x* represents a probability parameter, *y* is defined as a query, then the posterior probability p(y|e)(x) could be written as a fraction of two linear functions of *x* given the evidence *e*:

(13) $$p(y|e)(x)=\frac{\alpha_{1}x+\beta_{1}}{\gamma_{1}x+\delta }$$

The function could be normalized as following for simplicity:

(14) $$p(y|e)(x)=\frac{\alpha x+\beta }{\gamma x+1}$$

Message passing scheme in junction tree inference with *x*’s value set as 0, 0.5 and 1 is used in order to determine the value of α, β and γ:

(15) $$\{\begin{array}{c} \beta =p^{0}\\ \gamma =\frac{\beta -p^{0.5}}{p^{0.5}-p^{1}}-1\\ \alpha =p^{1}(\gamma +1)-\beta \end{array}$$

Then, the sensitivity value of query *y* at *x* given *e* can be obtained according to the partial derivative of p(y|e)(x) on *x*, which can be expressed as formula (16):

(16) $$f^{\prime }(x)=\frac{\partial p(y|e)}{\partial x}=\frac{\alpha -\beta \gamma }{{\left(\gamma x+1\right)}^{2}}$$

**Figure 9:**
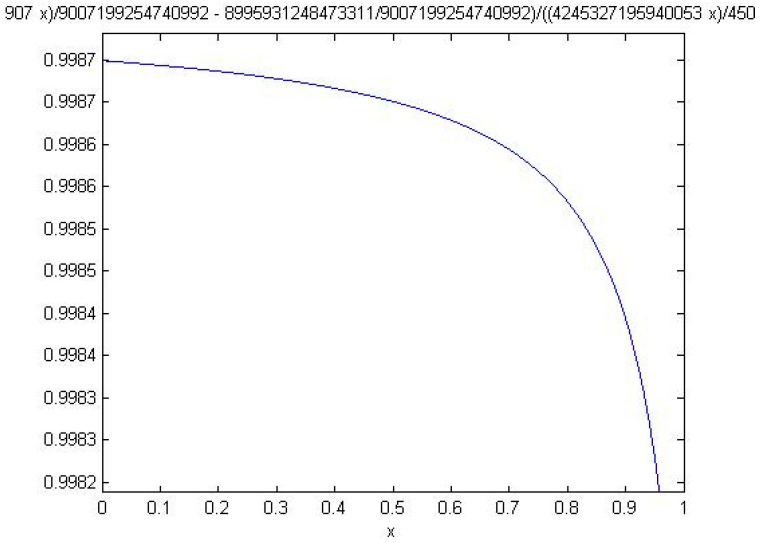
Sensitivity Function for the Node Crew Incapacitation.

In this paper, the node Crew Incapacitation is taken as an example to show the whole process. The sensitivity function of the node Crew Incapacitation is shown as follow:

(17) $$y=\frac{-0.941524x+0.998749}{-0.942652x+1}$$

where *y* represents the probability that flight crew performance is good and *x* stands for the probability of “Crew Incapacitation = yes”. The sensitivity function takes the form of curve in Figure 9.

Generally, the vertex of the curve could be found and it should be regarded as the turning point that divides the curve into two parts: in one part, the value of *y* changes sharply with the changes of *x*’s value (modulus of slope is greater than 1); and, the other part turns out to be inelastic (modulus of slope is less than 1). However, for the node Crew Incapacitation, vertex does not exist as modulus of slope for all *x*’s values are less than 1, indicating that the probability of crew incapacitation is inelastic (the probability of target node changes gently with the change of probability of crew incapacitation).

## 5. Conclusions

As a useful tool in solving problems with uncertainty and data scarcity, the Bayesian Network is applied to evaluate the flight crew performance. Important influence factors are selected based on the analysis of 484 aviation accidents that were caused by human factors. The Delphi technique helps to gather subjective data as a supplement to objective data from accident reports. CPTs are elicited by the leaky noisy MAX model. Two ways of inference for the BN—probability prediction and probabilistic diagnosis are used to analyze causal relations between factors and possible causes in the BN model.

Flying skills, vigilance, emergency mishandling, safety culture, and crew coordination are recognized as the most five important factors in flight crew performance.

Although the study shows the practical implementation of BN, the structure of BN model still needs to be modified according to additional practical feedback. Moreover, when it comes to probabilities distribution in risk analysis, the Delphi technique reflects weakness of validity. Other methods, such as the Classical Method, would be tried to improve the accuracy of CPTs when obtaining experts’ opinions.

## Figures and Tables

**Table 1:** Marginal Probabilities for Node Experience.

Pilot Flight Time (hours)	<1500	(1500, 5000)	≥5000
Number	12	73	349
State	poor	normal	rich
Probability	0.027650	0.168203	0.804147

**Table 2:** Computation Results of Changing States of Influence Factors.

Node	State	FCP ^1^ = good	State	FCP = good	State	FCP = good	Increased Percent
Flying Skills	poor	0.97263	qualified	0.99636	excellent	0.999247	2.74%
vigilance	low	0.98911	middle	0.997717	high	0.998753	0.97%
Emergency Mishandling	yes	0.993575	-	-	no	0.999217	0.57%
safety culture	poor	0.993667	normal	0.998564	good	0.999199	0.56%
Crew Coordination	poor	0.993972	normal	0.996798	good	0.999198	0.53%
training	poor	0.994111	normal	0.998586	good	0.999329	0.52%
Negligence or Misoperation	yes	0.993784	-	-	no	0.998802	0.50%
experience	poor	0.995181	middle	0.998638	rich	0.999167	0.40%
professional quality	poor	0.99589	middle	0.99855	good	0.999086	0.32%
organizational climate	poor	0.996428	normal	0.99832	good	0.999101	0.27%
communication	poor	0.996667	normal	0.998148	good	0.999153	0.25%
Violation	yes	0.99706	-	-	no	0.999305	0.23%
supervision	poor	0.996923	normal	0.998605	good	0.999012	0.21%
organization resources management	poor	0.997499	normal	0.998713	good	0.999113	0.16%
Judgment Error	yes	0.997443	-	-	no	0.998977	0.15%
operation plan	poor	0.997866	normal	0.998781	good	0.999149	0.13%
situational awareness	lose	0.997596	-	-	exist	0.998701	0.11%
Crew Incapacitation	yes	0.997864	-	-	no	0.998749	0.09%
physiological situation	poor	0.997872	normal	0.998696	good	0.99875	0.09%
working pressure	high	0.998308	middle	0.998827	low	0.998862	0.06%
mentation	poor	0.998226	normal	0.998676	good	0.998765	0.05%
policy	poor	0.998425	normal	0.998613	good	0.998827	0.04%
continuous working period	long	0.998531	-	-	normal	0.998752	0.02%
mood	poor	0.998605	normal	0.998691	good	0.998748	0.01%
circadian rhythms	drowsy	0.998657	-	-	awake	0.998745	0.01%
sudden injury	yes	0.998657	-	-	no	0.998745	0.01%

^1^ FCP represents flight crew performance for simplification.
