# Correction: Chen, W.; Huang, S.P. Evaluating Flight Crew Performance by a Bayesian Network Model. *Entropy* 2018, *20*, 178

**DOI:** 10.3390/e20120972

**Published:** 2018-12-14

**Authors:** Wei Chen, Shuping Huang

**Affiliations:** 1 School of Naval Architecture, Ocean and Civil Engineering, Shanghai Jiao Tong University, Shanghai 200240, China; 2 State Key Laboratory of Ocean Engineering, School of Naval Architecture, Ocean and Civil Engineering, Shanghai Jiao Tong University, Shanghai 200240, China

After publication of the research paper [1], the authors wish to make a correction to the authors’ affiliation. The address of the first author should be updated to “School of Naval Architecture, Ocean and Civil Engineering, Shanghai Jiao Tong University, Shanghai 200240, China”.

The manuscript will be updated and the original will remain online on the article webpage, with a reference to this correction. We apologize for any inconvenience caused by this mistake.
